# The Antinociceptive Potential of *Camellia japonica* Leaf Extract, (−)-Epicatechin, and Rutin against Chronic Constriction Injury-Induced Neuropathic Pain in Rats

**DOI:** 10.3390/antiox11020410

**Published:** 2022-02-17

**Authors:** Eun Yeong Lim, Changho Lee, Yun Tai Kim

**Affiliations:** 1 Division of Functional Food Research, Korea Food Research Institute, Wanju 55365, Korea; 50005@kfri.re.kr (E.Y.L.); chang@kfri.re.kr (C.L.); 2 Department of Food Biotechnology, Korea University of Science and Technology, Daejeon 34113, Korea

**Keywords:** *Camellia japonica*, (−)-epicatechin, rutin, neuropathic pain, analgesia

## Abstract

Neuropathic pain is caused by a lesion or disease of the somatosensory nervous system. Currently, prescribed treatments are still unsatisfactory or have limited effectiveness. *Camellia japonica* leaves are known to have antioxidant and anti-inflammatory properties.; however, their antinociceptive efficacy has not yet been explored. We examined the antinociceptive efficacy and underlying mechanism of *C. japonica* leaf extract (CJE) in chronic constriction injury (CCI)-induced neuropathic pain models. To test the antinociceptive activity of CJE, three types of allodynia were evaluated: punctate allodynia using von Frey filaments, dynamic allodynia using a paintbrush and cotton swab, and cold allodynia using a cold plate test. CCI rats developed neuropathic pain representing increases in the three types of allodynia and spontaneous pain. In addition, CCI rats showed high phosphorylation levels of mitogen-activated protein kinases (MAPKs), transcription factors, and nociceptive mediators in dorsal root ganglion (DRG). The ionized calcium-binding adapter molecule 1 levels and neuroinflammation also increased following CCI surgery in the spinal cord. CJE and its active components have potential antinociceptive effects against CCI-induced neuropathic pain that might be mediated by MAPK activation in the DRG and microglial activation in the spinal cord. These findings suggest that CJE, (−)-epicatechin, and rutin could be novel candidates for neuropathic pain management.

## 1. Introduction

Neuropathic pain is a chronic condition resulting from a lesion or disease of the somatosensory nervous system and is caused by various conditions, including strokes, multiple sclerosis, spinal cord injury, diabetic neuropathy, and postherpetic neuralgia [1,2]. The prevalence of neuropathic pain is estimated at 6.9–10% globally [3]. The clinical symptoms of neuropathic pain manifest as both stimulus-independent (spontaneous) and stimulus-dependent (evoked) pain [4]. Evoked pain includes hyperalgesia (abnormally increased pain sensation to a noxious stimulus) and allodynia (pain sensation to a non-noxious stimulus), which seriously affect a patient’s quality of life, incurring extreme economic burdens [5]. Currently prescribed pain relief treatments are still unsatisfactory or have limited effectiveness [6]. Therefore, many studies have attempted to elucidate the neuropathic pain mechanism and explore novel therapies for treating neuropathic pain.

The sensory neurons of the dorsal root ganglion (DRG) receive signals from peripheral nerve endings and integrate sensory signals into the spinal cord, and are implicated in the development and maintenance of neuropathic pain [7]. Maladaptive alterations associated with signaling pathways and transcription factors within the DRG after nerve injury affect hypersensitivity [8,9]. In addition, accumulating evidence indicates that inflammatory responses are involved in the pathogenesis of neuropathic pain [8,9]. Therefore, inhibition of the chronic constriction injury (CCI)-induced activation of signaling pathways, transcription factors, and inflammatory responses is a potential treatment strategy for peripheral neuropathy.

Microglia are prominent spinal cord glial cells that play pivotal roles in innate immune regulation and neuronal homeostasis in the central nervous system [10]. Accumulating evidence shows that neuroinflammation in the spinal cord resulting from glial cell activation is mainly related to neuropathic pain development, maintenance, and potentiation [11,12,13]. Following peripheral nerve injury, the anti-inflammatory microglial phenotype is converted to a proinflammatory phenotype in the spinal dorsal horn, releasing proinflammatory mediators that cause pain hypersensitivity [14]. Spinal microglial activation occurs in the early phase of neuropathic pain, followed by astrocytic activation, indicating that microglia may be essential for its initiation [15]. In addition, the inhibition of microglial activation by minocycline injection prevented the initiation of neuropathic pain in preclinical and clinical trials [16,17]. Therefore, targeting the inhibition of microglial activation and the release of proinflammatory mediators could be a potential therapeutic approach to prevent or resolve neuropathic pain.

Animal models of peripheral nerve injury help to elucidate neuropathic pain mechanisms and improve current clinical treatments. CCI is a widely used model of peripheral neuropathic pain proposed by Bennet and Xie and mimics the symptoms of chronic nerve compression in humans [18]. The rat model of CCI-induced neuropathic pain exhibited robust and long-lasting behavioral responses characterized by spontaneous pain, hyperalgesia, and allodynia [19]. CCI of the sciatic nerve evokes a series of changes in multiple genes and cellular pathways in the DRG and spinal cord [20,21]. Thus, the CCI model could assist in understanding neuropathic pain mechanisms and identifying novel analgesics.

Traditionally, various extracts of natural products have been used as analgesics. One candidate, *Camellia japonica*, has long been used in traditional herbal medicine to treat stomach disorders, bleeding, and inflammation, or as a tonic. The leaves of *C. japonica* show antioxidant [22], anti-inflammatory [23], and anti-hyperuricemic activities [24] and are composed of bioactive compounds, including triterpenes, flavonoids, and fatty acids [25,26]. Recently, the leaves of *C. japonica* were reported to contain high concentrations of vitamin E, n-eicosane, and rutin [24]. However, the antinociceptive efficacy of *C. japonica* leaves has not yet been explored. This study evaluated the analgesic effects of *C. japonica* leaf extract (CJE) and its active components and elucidated their mechanisms of action in CCI-induced neuropathic pain. We investigated abnormal changes, including the signaling pathways, transcription factors, and inflammatory mediators, induced by CCI surgery in the DRG and spinal cord.

## 2. Materials and Methods

### 2.1. Plant Material and Extraction

The leaves of *C. japonica* were collected from Taean-gun, Korea. The sample was identified by Dr. Jung-Hyun Lee and the voucher specimen (KFRI-MAT-0072) was deposited in the Food Functionality Research Group, Korea Food Research Institute, Wanju-gun, Korea. The washed leaves were dried overnight in a drying oven at 60 °C. The dried *C. japonica* leaves were blended using a mechanical grinder, and the powdered sample was immersed in 70% ethanol (10 times volume) at 80 °C for 6 h and then filtered using Whatman filter paper No. 1. The obtained filtrate was evaporated, and the samples were freeze-dried to obtain a dry extract. The CJE yield was calculated to be 16.7%.

### 2.2. Experimental Animals

All animal experiments complied with the Korea Food Research Institutional Animal Care and Use Committee guidelines (KFRI-M-19023). Male Sprague Dawley rats (6-weeks-old) were purchased from Orient Bio (Sung-Nam, South Korea) and randomly assigned to cages (two rats per cage). The rats were maintained under a controlled temperature (22 ± 2 °C) on a 12-h light/dark cycle with a standard laboratory diet and tap water ad libitum. The rats were allowed to acclimate for 1 week before the experiment. All efforts were made to minimize animal suffering and to reduce the number of animals used.

### 2.3. Chronic Constriction Injury (CCI) Surgery

The CCI model was induced using a previously described procedure, with minor modifications [18,27]. Briefly, the rats were anesthetized with isoflurane anesthesia (2% isoflurane mixed with oxygen), after which the left sciatic nerve was exposed at the mid-thigh level. Four loose 4-0 silk ligatures were tied around the nerve at 1 mm intervals at the proximal end to the sciatic trifurcation. A sham operation was performed with the exposure of the left sciatic nerve without ligation.

### 2.4. Experimental Design

The rats were randomly assigned to the 4 following groups to evaluate the antinociceptive effects of CJE against the CCI-induced neuropathic pain model: group I (sham; sham-operated rats received the vehicle (water) (p.o.)), group II (CCI with the vehicle; CCI-operated rats received the vehicle (p.o.)), group III (CCI with CJE; CCI-operated rats received 300 mg/kg of CJE (p.o.)), and group IV (CCI with pregabalin (PGB); CCI-operated rats received 30 mg/kg of PGB (p.o.)). The vehicle, CJE, and PGB were pre-treated for 1 week before CCI surgery and continued for 12 days.

Then, the rats were randomly assigned to 4 groups to determine the active ingredient of the CJE for pain relief: group I (sham; sham-operated rats received the vehicle (p.o.)), group II (CCI with the vehicle; CCI-operated rats received the vehicle (p.o.)), group III (CCI with (−)-epicatechin; CCI-operated rats received 30 mg/kg of (−)-epicatechin (p.o.)), and group IV (CCI with rutin; CCI-operated rats received 30 mg/kg of rutin (p.o.)). The vehicle, CJE, and PGB were pre-treated for 1 week before CCI surgery and continued for 15 days.

To evaluate the mechanism of analgesic effects, the rats were randomly assigned to five groups: group I (sham), group II (CCI with the vehicle), group III (CCI with CJE), group IV (CCI with (−)-epicatechin), and group V (CCI with rutin). All rats were sacrificed 3 days following surgery, and tissues were dissected.

### 2.5. Measurement of Pain-Associated Behavior

**Figure 1:**
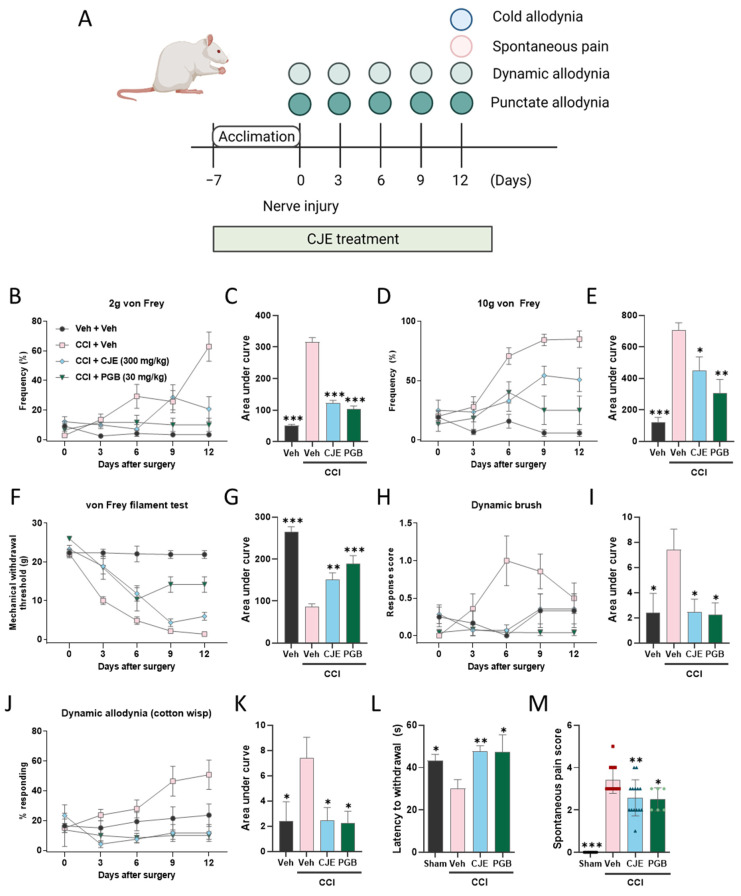
Effect of oral administration of *Camellia japonica* leaf extract (CJE) on pain sensitivity in a chronic constriction injury (CCI) model. (**A**) Experimental scheme of the pain-associated behavior test. Punctate allodynia was measured using the von Frey filament test. The paw withdrawal frequency elicited by (**B**,**C**) 2 and (**D**,**E**) 10 g von Frey filament stimulations. (**F**,**G**) The mechanical withdrawal threshold (MWT) was measured using a von Frey filament test. The area under the curve values for the paw withdrawal frequency elicited by 2 (**C**) and 10 g **(E)** von Frey filament stimulations, and the MWT (**G**). Dynamic allodynia was elicited using a dynamic brush test (**H**,**I**) and cotton wisp (**J**,**K**). The area under the curve values for the dynamic brush test (**I**) and cotton wisp (**K**). (**L**) Cold allodynia was measured using a cold plate test. (**M**) Spontaneous pain scores 12 days after CCI surgery. Black dots, red rectangles, blue triangles and green dots represent sham, CCI with the vehicle, CCI with CJE, and CCI with PGB, respectively. Data are presented as the mean ± SEM (*n* = 6–14) and analyzed using a one-way ANOVA by Dunnett’s post hoc test; * *p* < 0.05, ** *p* < 0.01, and *** *p* < 0.001 compared with CCI with vehicle treatment group.

The experimental scheme of the pain-associated behavior test is depicted in Figure 1A.

#### 2.5.1. Punctate Allodynia

##### Withdrawal Frequency Elicited by 2 and 10 g von Frey Filaments

Rats were placed on an elevated wire mesh grid in acrylic chambers and allowed to acclimate for at least 30 min. The von Frey filament (Stoleting, Chicago, IL, USA) was applied to the middle of the plantar surface of the hind paw with a 2 or 10 g filament. The withdrawal responses following hind paw stimulation were measured 10 times. The paw withdrawal response in each of these 10 trials was represented as the percent response frequency ((number of withdrawals/10 trials) × 100 = % response frequency), and this percentage was used to indicate punctate allodynia.

###### Mechanical Withdrawal Threshold (MWT)

The MWT was measured using von Frey filaments, as previously reported [28]. After acclimation, the MWT was determined using a series of von Frey filaments applied to the middle of the plantar surface of the hind paw. Testing was initiated with the 2 g filament, and the threshold was determined using the up–down method. Once a positive withdrawal response was established, the strength of the next filament was decreased, and if the animal did not respond, the strength of the next filament was increased. A positive response was recorded if the paw was sharply withdrawn or shaking, licking, or flinching upon application of the filament.

#### 2.5.2. Dynamic Allodynia

##### Brush-Evoked Dynamic Allodynia

Dynamic allodynia was measured using a paintbrush as previously described [29,30]. The rats were acclimated in the von Frey chambers for 30 min, and a paintbrush was gently stroked along the lateral side of the rat’s hind paw from the heel to the toe. The response was scored as previously described [30]: 0, no response; 1, very short, fast movement/lifting of the paw; 2, sustained lifting of the paw for more than 2 s toward the body or pronounced lateral lifting above the body level; and 3, flinching, licking, or flicking the affected paw. The allodynia scores were reported as the average scores across 3 trials per rat.

##### Cotton Swab Assay

Dynamic responses were assessed by stroking a cotton swab along the plantar surface of the rat’s hind paw, as described previously, with slight modifications [31,32]. The rats were acclimated for 30 min in the von Frey chambers. A cotton swab was puffed out to 3 times its original size, and 10 sweeps were performed with at least 10 s between each trial. Positive responses were defined as withdrawal or rapid paw shaking. The number of withdrawals was counted and reported as the percentage withdrawal for each rat.

#### 2.5.3. Cold Allodynia

The paw withdrawal latency was measured with a hot/cold plate analgesia meter (IITC Life Science, Woodland Hills, CA, USA) set at 0 °C according to previously described methods [33]. The latency to nocifensive behavior (e.g., licking, biting, shaking, or lifting the hind paws) was recorded. The cut-off time was 60 s to prevent tissue damage.

#### 2.5.4. Spontaneous Pain-Related Behaviors

To assess spontaneous pain, the rats were acclimated in the von Frey chambers for 30 min, and spontaneous pain-related behavior was observed for 5 min. The response was scored as described previously [34,35]: 0, the paw of the operated side was pressed normally on the floor; 1, the paw rested lightly on the floor; 2, only the paw’s inner edge was pressed on the floor; 3, only the paw’s heel was pressed on the floor and the hind paw was inverted; 4, the whole paw was elevated; and 5, the animal licked the ipsilateral paw. The largest observed scale number was adopted to estimate the response score.

### 2.6. High-Performance Liquid Chromatography (HPLC) Analysis

HPLC analysis was conducted using a Jasco HPLC system (Jasco, Tokyo, Japan) equipped with dual pumps, an autosampler, a column oven, and a detector. Chromatographic separations were carried out on a Waters Symmetry C18 column (particle size 5 μm, 4.6 × 250 mm) and monitored at 280 nm, with the temperature maintained at 35 °C. The injection volume was 10 μL, and the flow rate was 0.6 mL/min. CJE and the standard mixture were dissolved in 50% methanol. Reverse-phase HPLC assays were performed by gradient elution using a mobile phase comprising of (A) 0.5% formic acid and (B) acetonitrile:methanol (80:20 *v*/*v*), as follows: 0–30 min from 5–35%, 30–36 min from 35–90%, and 36–45 min from 90–5% elution gradient using solvent B. This condition was maintained for 5 min. The standard calibration curves for (−)-epicatechin, rutin, hyperoside, and isoquercitrin were linear (r^2^ > 0.999 **) in the range of 40–200 μg/mL. Analyses were performed in triplicate.

### 2.7. Western Blotting

Western blotting was performed as previously described, with slight modifications [36]. The rats were euthanized under isoflurane anesthesia, and the L4–L6 DRG and spinal cord were isolated by hydraulic extrusion [37]. Briefly, homogenates were loaded onto sodium dodecyl sulfate–polyacrylamide gel electrophoresis and were transferred to polyvinylidene fluoride membranes. The membranes were blocked in 5% skim milk in tris-buffered saline with 0.1% Tween 20 (TBST) and incubated overnight at 4 °C with primary antibodies against p-ERK (Cell Signaling Technology, CST, Danvers, MA, USA), ERK (CST), p-p38 (CST), p-38 (CST), p-JNK (CST), JNK (CST), matrix metallopeptidase 9 (MMP9) (Abcam, Cambridge, MA, USA), C-C chemokine receptor type 2 (CCR2) (Novus Biologicals, Littleton, CO, USA), CX3C chemokine receptor 1 (CX3CR1) (Abcam), and α-Tubulin (CST). After washing 3 times for 5 min with TBST, the membranes were incubated with secondary antibodies (Bethyl Laboratories, Montgomery, TX, USA) for 1 h at room temperature. After washing, the membranes were visualized using enhanced chemiluminescence (Thermo Fisher Scientific, Rockford, IL, USA). The band density was measured and normalized against the vehicle band.

### 2.8. Total RNA Isolation and Quantitative Real-Time Polymerase Chain Reaction (qRT-PCR) Assays

The total RNA was extracted using the NucleoPin^®^ RNA XS Kit (Macherey-Nagel, Bethlehem, PA, USA) according to the manufacturer’s instructions. After RNA quantification on the nanodrop, reverse RNA transcription was performed using ReverTra Ace^®^ qPCR RT Master Mix (Toyobo, Osaka, Japan), and qRT-PCR was performed using Power SYBR^®^ Green PCR Master Mix (Applied Biosystems, Carlsbad, CA, USA). Thermal cycling was carried out using a QuantStudio 6 real-time PCR system (Applied Biosystems, Foster City, CA, USA). The relative quantitative mRNA was normalized using the housekeeping glyceraldehyde 3-phosphate dehydrogenase gene using the 2^−∆∆CT^ method. The specific primer pairs used are listed in Table 1.

### 2.9. Immunofluorescence

The rats were euthanized under isoflurane anesthesia, and the L4–L6 DRG and spinal cord were isolated by hydraulic extrusion [37]. The isolated tissue was post-fixed with 4% paraformaldehyde at 4 °C overnight. The paraformaldehyde was replaced with 15 and 30% sucrose for the cryoprotection of the DRG and spinal cord, respectively, and the tissue was incubated overnight at 4 °C until the tissue sank. The tissue was embedded in an optimal cutting temperature (OCT) compound and cryosectioned at thicknesses of 20 and 30 µm for the DRG and spinal cord, respectively, for immunostaining.

The sections were first washed in phosphate buffer solution (PBS) to remove the excess OCT compound and blocked in 10% normal goat serum in PBS with 0.1% Triton X-100 (PBST) for 1 h at room temperature. The sections were then incubated with primary antibodies overnight at 4 °C. The following day, the sections were washed and then incubated in darkness with a secondary antibody for 2 h at room temperature. After washing with PBST, the sections were mounted with DAPI Fluoroshield (Sigma–Aldrich, St. Louis, MO, USA) and imaged. Fluorescence images were captured using a laser scanning confocal microscope (FV3000, Olympus Corporation, Tokyo, Japan), and the fluorescence intensity was analyzed using ImageJ software.

### 2.10. Statistics

Statistical analysis was performed using GraphPad Prism version 9 (GraphPad Software, San Diego, CA, USA). The results are expressed as the mean ± standard error of the mean (SEM). Statistical significance was evaluated using an unpaired *t*-test for between-group comparisons and one-way analysis of variance followed by Dunnett’s post hoc test for multiple group comparisons. Statistical significance was set at *p* < 0.05.

## 3. Results

### 3.1. C. japonica Leaf Extract Attenuates Chronic Constriction Injury-Induced Pain Sensitivity

To investigate the antinociceptive effects of CJE on CCI-induced pain sensitivity, three types of allodynia (punctate, dynamic, and cold) and spontaneous pain behavior were evaluated. Punctate allodynia was measured using the von Frey filament test (Figure 1B–G). At baseline, there were no significant between-group differences. Following CCI, the pain response to punctate allodynia was increased in CCI rats, with increased frequency elicited by 2 and 10 g von Frey filament stimulation (Figure 1B–E) and decreased MWT in the von Frey filament test (Figure 1F,G). In this study, PGB served as a positive control, and treatment of PGB also reduced the CCI-induced pain sensitivity (Figure 1B–M). Pretreatment of CJE significantly decreased the response frequency to 2 and 10 g von Frey filament stimulation as compared with the CCI group (Figure 1B–E). In addition, decreased MWT was reversed in the CCI with CJE treatment group at 3–12 d post-surgery (Figure 1F,G). These results suggest that the oral administration of CJE dramatically reduced punctate allodynia in CCI rats.

To test whether CJE treatment ameliorated CCI-induced dynamic allodynia, paintbrush and cotton swab tests were performed. The basal dynamic mechanical allodynia did not differ between the groups (Figure 1H–K). The CCI groups exhibited a significant increase in the dynamic allodynia scores and response frequency. The dynamic allodynia score was lower in the CCI with CJE treatment group than that in the CCI with vehicle treatment group (Figure 1H–K).

CCI resulted in a significant decrease in the withdrawal latency on the cold plate, and this decreased latency was reversed in the CJE-treated group (Figure 1L). The spontaneous pain behavior scores were significantly increased 12 days postoperatively; CJE treatment inhibited the pain behavior score when compared with the CCI with vehicle treatment group (Figure 1M).

### 3.2. (−)-Epicatechin and Rutin Attenuates Chronic Constriction Injury-Induced Pain Sensitivity

**Figure 2:**
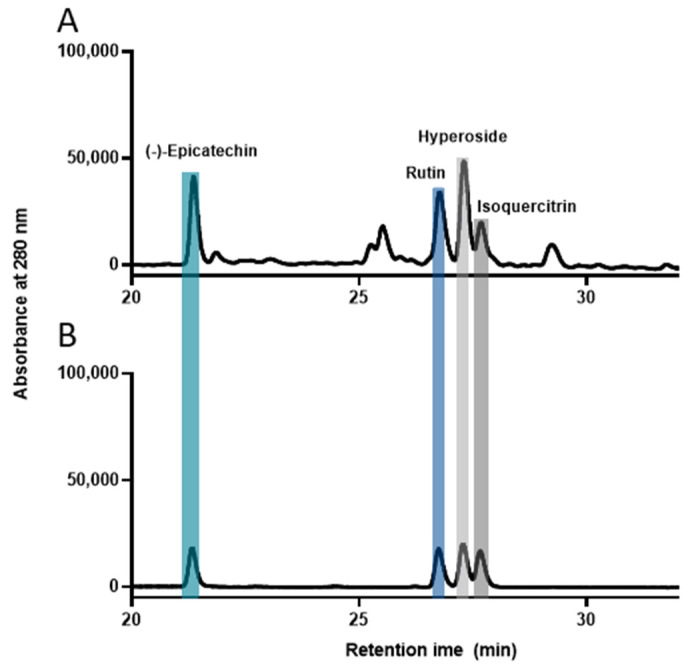
Chemical compounds of *Camellia japonica* leaf extracts (CJE). (**A**) High-performance liquid chromatography chromatogram of CJE and (**B**) standard mixture at a wavelength of 280 nm.

HPLC was performed to identify the components of CJE. (−)-Epicatechin, rutin, hyperoside, and isoquercitrin were identified in the HPLC chromatogram of CJE at retention times of 21.4, 26.8, 27.3, and 27.7 min, respectively. The concentrations were 10.53 ± 0.06, 44.41 ± 0.38, 33.13 ± 0.23, and 28.51 ± 0.41 mg/g, respectively. The chromatograms of the CJE and standard mixtures are shown in Figure 2. 

**Figure 3:**
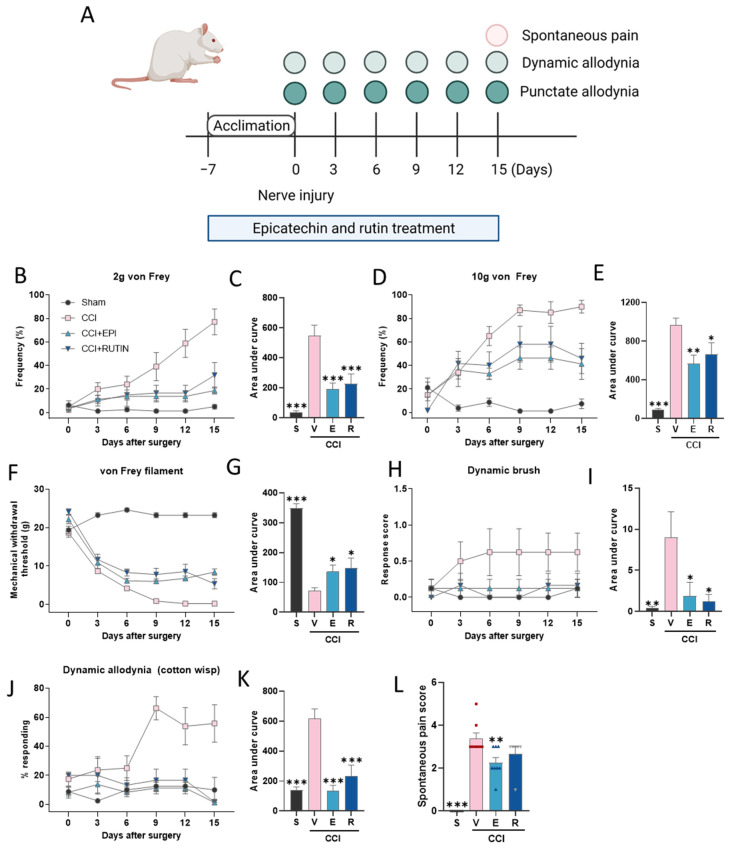
Effect of oral administration of (−)-epicatechin and rutin on pain sensitivity in a chronic constriction injury (CCI) model. (**A**) Experimental scheme of the pain-associated behavior test. Punctate allodynia was measured using a von Frey filament. The paw withdrawal frequency under 2 (**B**,**C**) and 10 g (**D**,**E**) von Frey filament stimulations. (**F**,**G**) The mechanical withdrawal threshold (MWT) was measured using the von Frey filament test. The area under the curve values for the paw withdrawal frequency under 2 (**C**) and 10 g (**E**) von Frey filament stimulations, and the MWT (**G**). Dynamic allodynia was elicited using a dynamic brush test (**H**,**I**) and cotton wisp (**J**,**K**). The area under the curve values for the dynamic brush test (**I**) and cotton wisp (**K**). (**L**) Spontaneous pain scores 15 days after CCI surgery. Black dots, red rectangles, blue triangles and gray triangles represent sham, CCI with the vehicle, CCI with (−)-epicatechin, and CCI with rutin, respectively. Data are presented as the mean ± SEM (*n* = 6–8) and analyzed using one-way ANOVA by Dunnett’s post hoc test; * *p* < 0.05, ** *p* < 0.01, and *** *p* < 0.001 compared with CCI with the vehicle treatment group.

Punctate and dynamic allodynia were evaluated every 3 days during the experimental periods and spontaneous pain behavior was measured 15 days after surgery (Figure 3A). The antinociceptive effects of (−)-epicatechin and rutin were evaluated to determine the active pain-relieving components of CJE. Treatment with (−)-epicatechin and rutin reduced CCI-induced punctate allodynia, resulting in high MWT and low frequency elicited by 2 and 10 g von Frey filament stimulation compared with the CCI group (Figure 3B–G). In addition, (−)-epicatechin and rutin attenuated dynamic allodynia (Figure 3H–K) and the spontaneous pain behavior score (Figure 3L).

### 3.3. C. japonica Leaf Extract, (−)-Epicatechin, and Rutin Inhibit the Chronic Constriction Injury-Induced Upregulation of MAPKs Phosphorylation in the Dorsal Root Ganglion

**Figure 4:**
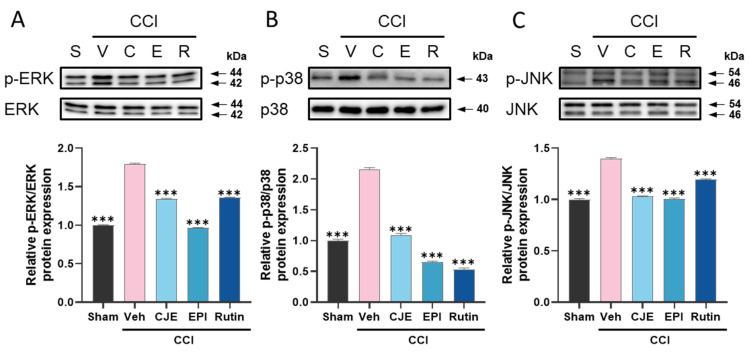
Effect of oral administration of CJE, (−)-epicatechin, and rutin on the mitogen-activated protein kinases (MAPKs) signaling pathway in the dorsal root ganglion (DRG) in chronic constriction injury model. (**A**–**C**) Representative Western blots and quantitative Western blot analysis of MAPKs expression were performed in L4–L5 DRG tissues. Representative Western blots and quantitative analysis of the protein levels of phosphorylated extracellular signal-regulated kinase (ERK)1/2 (**A**), p38 (**B**), and c-Jun N-terminal kinase (JNK) (**C**) in the DRG. Data are presented as the mean ± SEM of three independent experiments and analyzed using one-way ANOVA; *** *p* < 0.001 compared with CCI with the vehicle treatment group.

To determine whether the analgesic effects of CJE on the CCI-induced neuropathic pain model were associated with inhibition of the MAPK signaling pathway, we evaluated the phosphorylation of MAPK in the DRG using Western blot analysis. At 3 days after CCI surgery, MAPKs (ERK, p38, and JNK) phosphorylation increased as compared with that in the sham group. Oral administration of CJE inhibited the upregulation of the phosphorylation of ERK, p38, and JNK in the DRG of rats compared with that in rats from the CCI with vehicle treatment group (Figure 4A–C). These results suggest that CJE, (−)-epicatechin, and rutin may have analgesic effects by inhibition of MAPKs against CCI-induced peripheral neuropathic pain.

### 3.4. C. japonica Leaf Extract and Rutin Inhibit the Chronic Constriction Injury-Induced Increase in the ATF3 and c-Jun Expression in the Dorsal Root Ganglion 3 Days after Chronic Constriction Injury in Rats

**Figure 5:**
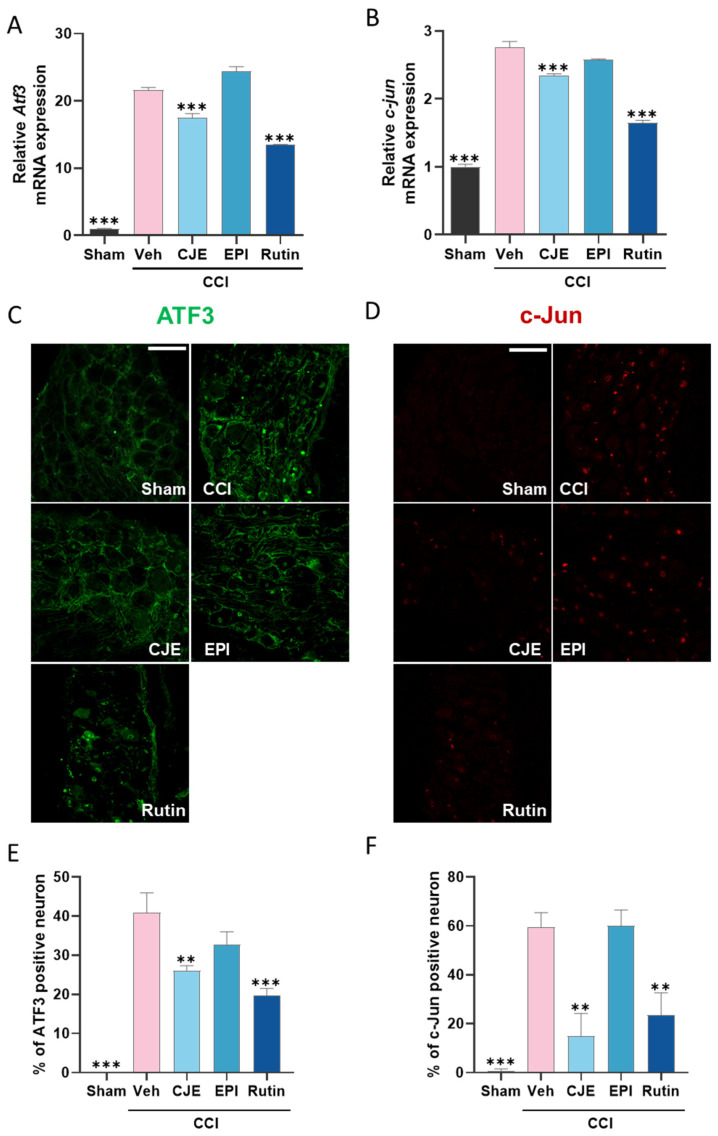
Effect of oral administration of CJE, (−)-epicatechin, and rutin on transcription factors in the dorsal root ganglion in a chronic constriction injury model. The mRNA expression of activating transcription factor 3 (*Atf3*) (**A**) and *c-jun* (**B**) was detected by the real-time polymerase chain reaction (qRT-PCR). Representative fluorescence images for ATF3 (**C**) and c-Jun (**D**). The bars represent the neurons positive for ATF3 (**E**) and c-Jun (**F**). The scale bar denotes 100 µm. Data are presented as the mean ± SEM of four independent experiments and analyzed using one-way ANOVA; ** *p* < 0.01, and *** *p* < 0.001 compared with CCI with the vehicle treatment group.

Transcription factors are involved in the development and maintenance of neuropathic pain. The qRT-PCR results showed that the mRNA expression of *Atf3* and *c-jun* increased in the ipsilateral DRG of CCI rats (Figure 5A,B). Although (−)-epicatechin did not affect the expression of *Atf3* and *c-jun*, CJE and rutin inhibited the expression *of Atf3* and *c-jun* when compared with that in the CCI group. Immunohistochemistry analysis also confirmed that the ATF3 and c-Jun protein levels were increased in the ipsilateral DRG, blocked by the CJE and rutin treatment (Figure 5C–F).

### 3.5. C. japonica Leaf Extract, (−)-epicatechin, and Rutin Inhibit the Chronic Constriction Injury-Induced Increase in Inflammatory Mediators in the Dorsal Root Ganglion 3 Days after Chronic Constriction Injury in Rats

**Figure 6:**
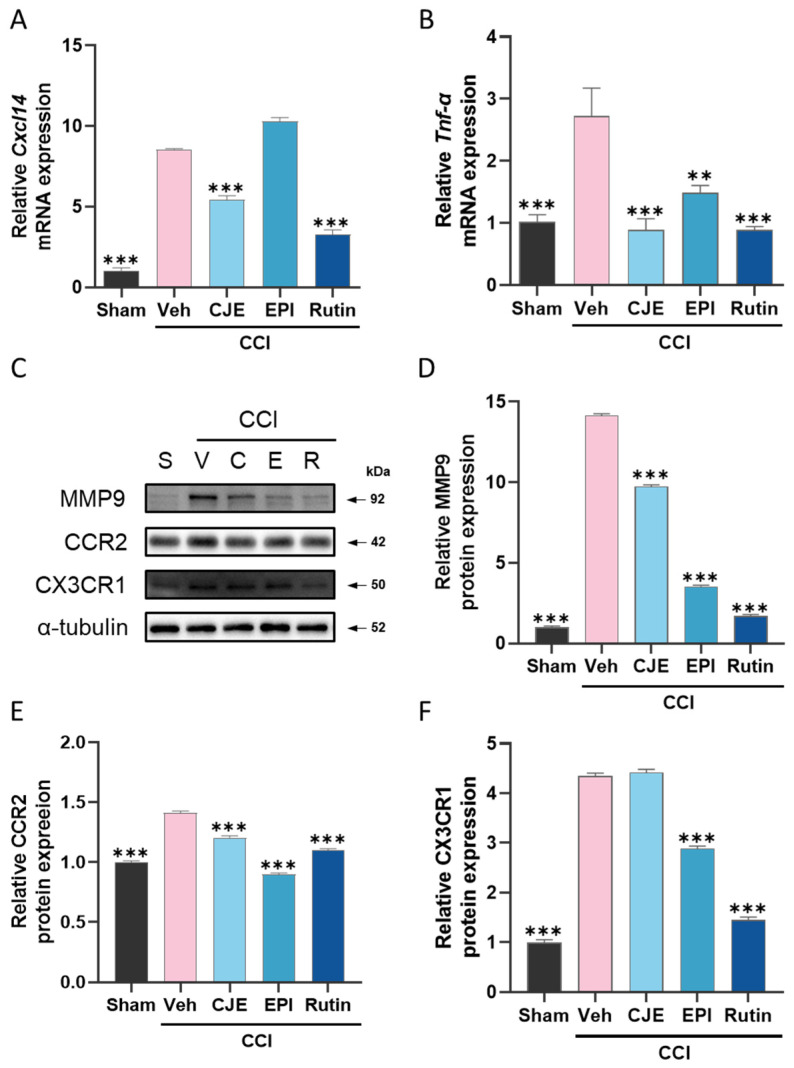
Effect of oral administration of CJE, (−)-epicatechin, and rutin on inflammatory mediators in the dorsal root ganglion (DRG) in a chronic constriction injury model. The mRNA expression of chemokine ligand 14 (*Cxcl14*) (**A**) and tumor necrosis factor α (*Tnf-α*) (**B**) were detected by qRT-PCR in the DRG. Representative Western blots of the protein levels of matrix metallopeptidase 9 (MMP9), C-C chemokine receptor type 2 (CCR2), and CX3C chemokine receptor 1 (CX3CR1) in the DRG (**C**). Quantitative analysis of the protein levels of MMP9 (**D**), CCR2 (**E**), and CX3CR1 (**F**). Data are presented as the mean ± SEM of three and four independent experiments for mRNA and protein expression, respectively, and analyzed using one-way ANOVA; ** *p* < 0.01 and *** *p* < 0.001 compared with CCI with the vehicle treatment group.

To evaluate the pronociceptive mediator change in the DRG following CCI surgery, we performed qRT-PCR and Western blotting. qRT-PCR analysis showed that tumor necrosis factor α (*Tnf-α*) and chemokine ligand 14 (*Cxcl14*) mRNA expression increased in the ipsilateral DRG in the CCI rats (Figure 6A,B). CJE, (−)-epicatechin, and rutin reduced the increase in the mRNA expression of *Tnf-α*, and CJE and rutin also reduced the *Cxcl14* mRNA expression in the DRG, but (−)-epicatechin did not (Figure 6A,B). As measured by Western blotting, our results showed that CCI induced a significant upregulation of pronociceptive genes, such as MMP-9, CCR2, and CX3CR1 (Figure 6C–F). (−)-epicatechin and rutin treatment showed an inhibitory effect on MMP-9, CCR2, and CX3CR1; CJE treatment inhibited the MMP-9 and CCR2 expression levels (Figure 6C–F).

### 3.6. C. japonica Leaf Extract, (−)-Epicatehin, and Rutin Inhibit Microglial Activation in the Spinal Dorsal Horn

**Figure 7:**
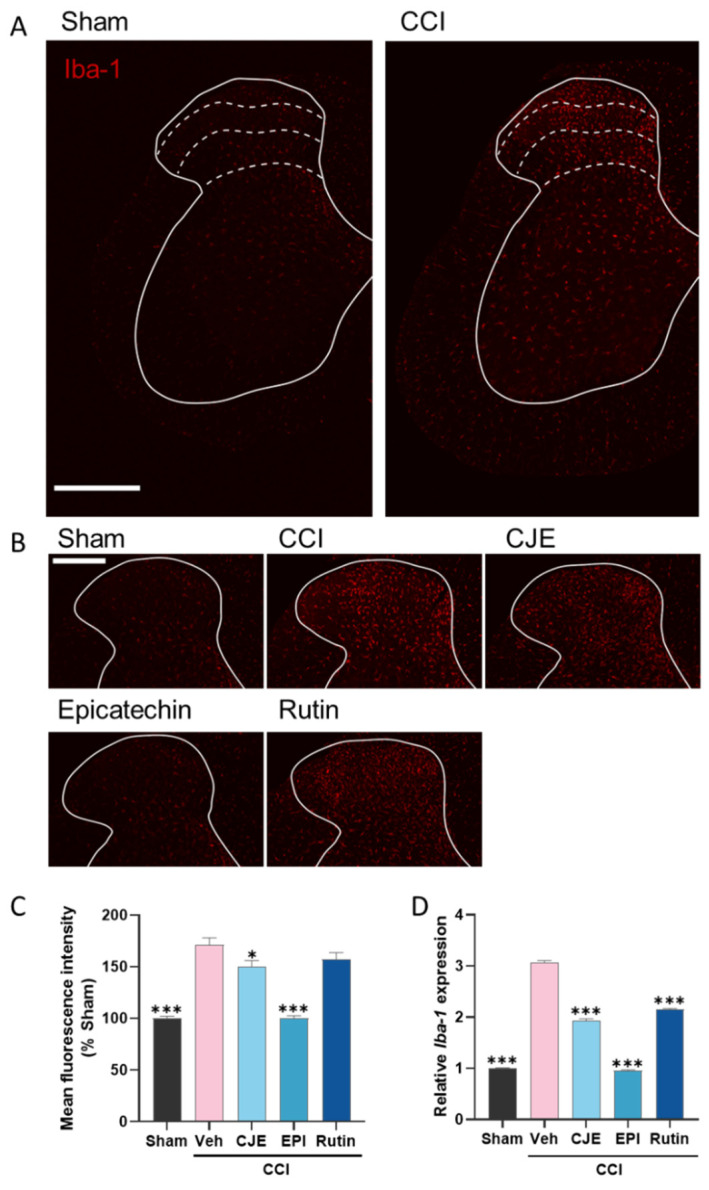
Effect of oral administration of CJE, (−)-epicatechin, and rutin on the microglial marker, ionized calcium-binding adaptor molecule allograft inflammatory factor 1 (Iba-1), in the spinal cord in the CCI model. (**A**) Representative confocal immunofluorescence images of the ipsilateral dorsal horn of the spinal cord immunostaining of Iba-1 in the sham and CCI groups 3 days postoperatively. The scale bar denotes 500 µm. (**B**) Magnification of the confocal immunofluorescence images of the ipsilateral dorsal horn of the spinal cord immunostaining of Iba-1 in the sham, CCI, CJE, (−)-epicatechin, and rutin treatment groups. The scale bar denotes 300 µm. (**C**) Quantification of the mean fluorescence intensity of Iba-1. (**D**) The mRNA expression of *Iba-1* was detected by qRT-PCR in the DRG. Data are presented as the mean ± SEM of three independent experiments and analyzed using one-way ANOVA; * *p* < 0.05, *** *p* < 0.001 compared with CCI with the vehicle treatment group.

Microglial activation is involved in the development of neuropathic pain. Since pretreatment with CJE and its active compounds improved the neuropathic behavior of the CCI model, we focused on the microglia. We evaluated the effect of CJE, (−)-epicatechin, and rutin on CCI-induced microglial activation in the spinal dorsal horn using immunohistochemistry and qRT-PCR. Immunohistochemical analysis showed that ionized calcium-binding adaptor molecule 1 (Iba-1), a marker of microglia, was increased in the ipsilateral dorsal horn of CCI rats compared to that in sham rats (Figure 7A). As shown in Figure 7B,C, compared with the CCI group, CJE and its active compounds significantly decreased CCI-induced Iba-1 expression. Specifically, (−)-epicatechin treatment showed a significant reduction in Iba-1 expression, with expression levels similar to those in the sham group (Figure 7B,C). Similar to the immunohistochemistry data, we confirmed the above results using qRT-PCR analysis (Figure 7D). These results suggest that CJE, (−)-epicatechin, and rutin attenuate pain sensitivity in the CCI model by suppressing spinal microglial activity.

### 3.7. C. japonica Leaf Extract, (−)-Epicatehin, and Rutin Inhibits Inflammatory Mediator Expression in the Spinal Cord

**Figure 8:**
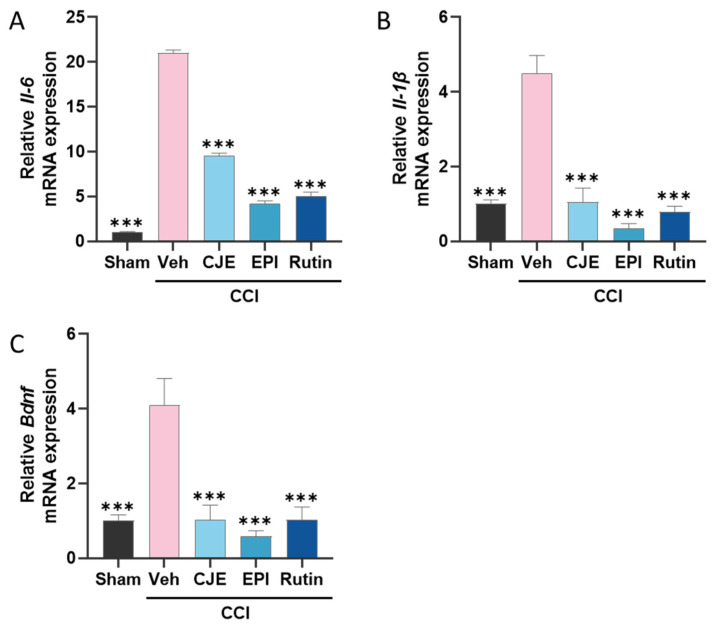
Effect of oral administration of CJE, (−)-epicatechin, and rutin on neuroinflammation in the spinal cord in the chronic constriction injury model. The mRNA expressions of Interleukin-6 (*Il-6*) (**A**), *Il-1β* (**B**), and brain-derived neurotrophic factor (*Bdnf*) (**C**) were detected by qRT-PCR. Data are presented as the mean ± SEM of four independent experiments and analyzed using one-way ANOVA; *** *p* < 0.001 compared with CCI with the vehicle treatment group.

Activated microglia release a variety of pronociceptive genes contributing to neuropathic pain maintenance. To confirm the inhibitory effects of CJE and its active compounds on the pain-related genes, the mRNA expression of interleukin 6 (*Il-6*) and *Il-1β*, and brain-derived neurotrophic factor (*Bdnf*) expression were measured in the spinal cords of rats with neuropathic pain. Following CCI surgery, there was a significant increase in the inflammatory mediator levels in *Il-6*, *Il-1β*, and *Bdnf* compared with those in the sham group. Compared with the CCI group, the CCI with CJE, (−)-epicatechin, and rutin treatment groups showed a significant decrease in the expression of inflammatory mediators in the spinal cord (Figure 8A–C).

## 4. Discussion

This study evaluated the antinociceptive effects of CJE and determined its active components: (−)-epicatechin and rutin. Treatment with CJE, (−)-epicatechin, and rutin attenuated several types of allodynia, including punctate, dynamic, and cold allodynia, and spontaneous pain. Further analysis indicated that CJE, (−)-epicatechin, and rutin reversed the activation of MAPKs and inflammatory or nociceptive mediators in the DRG. Furthermore, CJE and rutin treatment reduced the transcription factor in the DRG. In addition, CJE, (−)-epicatechin, and rutin administration inhibited microglial activation and neuroinflammation in the spinal cord. These results demonstrate that CJE, (−)-epicatechin, and rutin treatment could be used as a novel alternative therapy for neuropathic pain.

We used a CCI-induced neuropathic pain model, a well-recognized neuropathic pain model that helps to elucidate pain mechanisms, which successfully develops pain manifestations, including mechanical and thermal allodynia, and exhibits spontaneous pain similar to clinical phenomena. CJE, (−)-epicatechin, and rutin showed significant attenuation in these types of allodynia and spontaneous pain in the ipsilateral paw in the CCI model.

Flavonoids are the most common secondary plant metabolites and have analgesic effects on pain sensitivity. Considering several studies that have been conducted that found that antioxidants could relieve neuropathic pain [38], flavonoids have the potential to relieve pain sensitivity, because they are powerful antioxidants [39]. In particular, (−)-epicatechin and rutin are potent antioxidant agents in vitro and in vivo [40,41]. (−)-epicatechin alleviated pain hypersensitivity in a glutamate-induced nociception model [42] and showed antinociceptive effects on formalin- and carrageenan-induced inflammatory pain, and spinal nerve ligation-induced neuropathic pain [43]. In addition, (−)-epicatechin showed antinociceptive effects in a diabetes-induced painful model [44]. Rutin also has been reported to have analgesic efficacy against streptozotocin- and oxaliplatin-induced neuropathic pain [45,46]. This study demonstrated that (−)-epicatechin and rutin attenuated CCI-induced neuropathic pain and confirmed their pain relief efficacy. The HPLC chromatograms of CJE revealed that isoquercitrin and hyperoside are components of CJE. The potent anti-allodynic effects of CJE could be demonstrated by the synergistic interactions between these flavonoids.

The DRG consists of a bundle of nerve cells located in the dorsal root of the spinal nerve and transmits pain signals from the peripheral nerves to the spinal cord. The complex molecular cascade response to peripheral nerves in the DRG contributes to spontaneous action potential activity and increased neurotransmitter release. Maladaptive changes in nociceptors, ion channels, immune cell activation, inflammation, and signaling pathways are involved in delivering noxious stimuli following nerve injury [7,47]. The regulation of these genes in DRG may provide a new approach for neuropathic pain management. Consequently, we investigated the CCI-induced changes in the MAPK signaling pathway, transcription factors, and nociceptive mediators within the DRG to identify CJE, (−)-epicatechin, and rutin’s anti-allodynic effects.

MAPKs, including ERK, JNK, and p38, are crucial in intracellular signaling pathways, such as cell differentiation, proliferation, and apoptosis in response to various stimuli [48]. Many studies have revealed that MAPKs are activated in several neuropathic pain models of the DRG [49,50], and they are known to synthesize various inflammatory mediators, including TNF-α and IL-1β, subsequently contributing to neuroinflammation and neuropathic pain. Indeed, the DRG of CCI models showed high MAPK expression, and the inhibition of these elevated expressions could attenuate neuropathic pain [51]. Thus, the inhibition of MAPK signaling in the DRG may provide a promising strategy for developing novel analgesics. In this study, we exhibited that CJE and its active components significantly inhibited MAPK activation in DRG.

Several transcription genes are elevated and activated in the DRG following peripheral nerve injury [52]. ATF3 has been implicated as a marker of nerve injury and can be characterized as an ‘adaptive response’ gene for cells to cope with extra-and intracellular changes. ATF3 expression is upregulated in chemotherapy-and surgery-induced neuropathic pain [53,54], and ATF3-deletion mice showed decreased mechanical allodynia in diabetic peripheral neuropathy [55]. Another transcription factor, c-Jun, the best-known substrate of JNK, was considered as a hub protein and a key gene that may play an important role in the development of neuropathic pain. The activation of c-Jun in the DRG contributes to the pathogenesis of neuropathic pain [56], and intrathecal injection with c-Jun antisense oligodeoxynucleotides significantly reduced ipsilateral mechanical allodynia in CCI models [56]. Deleting dual leucine zipper kinase reduces the injury-induced transcription factors phosphorylated-c-Jun and ATF3 in the DRG following the spared nerve injury and attenuates neuropathic pain [57]. Our results indicate that treatment with CJE and rutin reduced the CCI-induced expression of ATF3 and c-Jun in the DRG.

The development of neuropathic pain involves the peripheral immune system and many different cell types, including DRG, Schwann, and satellite glial cells. These cells release inflammatory mediators following nerve injury and further potentiate neuropathic pain. Inflammatory mediators include cytokines (TNF-α, IL-1β, and IL-6), chemokines (CCL2 and CX3CL1), and proteases (MMP-9 and cathepsin S) [58]. TNF-α expression was increased in the early phase of a CCI-induced neuropathic pain model [59]. TNF-α silencing or inhibition attenuated mechanical allodynia in neuropathic pain models [60,61]. Additionally, CXCL14 is known to be upregulated in neuropathic pain models, and decreased levels of CXCL14 are accompanied by pain relief [62,63]. MMP-9 is rapidly and transiently upregulated in injured DRG primary sensory neurons, consistent with the early phase of neuropathic pain, and the intrathecal injection of MMP-9 is sufficient to produce neuropathic pain symptoms [64].

In addition to chemokines, chemokine receptors are also implicated in neuropathic pain. The CCR2 levels in the DRG significantly increased in oxaliplatin- and CCI-induced neuropathic pain, and blocking CCL2/CCR2 signaling attenuated neuropathic pain [65,66]. The fractalkine receptor, CX3CR1, a marker of peripheral monocytic cells and microglia, was increased in the ipsilateral DRG in a spared nerve injury-induced neuropathic pain model [67]. In addition, the involvement of MMP-9 in the early phase of neuropathic pain in CCI rats was related to the upregulation of C-X3-C Motif Chemokine Ligand 1 (CX3CL1)/CX3CR1 [68]. Our results showed that CJE, (−)-epicatechin, and rutin treatment significantly reduced the upregulation of gene expressions. These findings suggest that CJE and its active components’ analgesic effects in neuropathic pain are mediated by the downregulation of CCI-induced nociceptive mediators.

Microglia are activated by proinflammatory mediators released by neurons or glial cells following nerve injury, and activated microglia can further release these mediators, creating a feedback loop that contributes to neuropathic pain. Specifically, spinal microglia are primarily activated in the early phase of neuropathic pain, and the inhibition of microglia attenuates the initiation of neuropathic pain. Consistent with our research, microglia were activated 3 days after CCI surgery, while astrocytes were not activated [69,70]. Our results showed that CJE, (−)-epicatechin, and rutin treatment significantly inhibited the activation of microglia and pro-inflammatory cytokines in the spinal cord. These mechanisms might contribute to CJE’s analgesic effects in our neuropathic pain model. Specifically, (−)-epicatechin showed a potent inhibitory effect on spinal microglia activation, similar to that of the sham-operated group.

Neuroinflammation in the spinal cord is associated with the development and maintenance of neuropathic pain. Following nerve injury, activated microglia released proinflammatory cytokines (e.g., TNF-α, IL-1β, and IL-6) and BDNF. These genes have been strongly involved in the initiation and development of neuropathic pain after nerve injury. Proinflammatory cytokines and BDNF sensitize dorsal horn neurons and cause persistent pain states. IL-6 has been shown to promote pain conditions, and beneficial effects are achieved through the intrathecal injection of the blocking antibodies or antagonists. Intrathecal injection of IL-1β increased dorsal horn neuron excitability and produced mechanical allodynia [71]. Genetic impairment of IL-1β signaling attenuates neuropathic pain [72]. Activated microglia in the spinal cord released the BDNF, which increased dorsal horn excitability. Increased BDNF expression in the spinal dorsal horn is associated with the progress of central sensitization and pain. The inhibition of the expression of proinflammatory factors can alleviate mechanical allodynia and thermal hyperalgesia in a chronic constriction injury (CCI) rat model. In the present study, CJE and its active compounds significantly inhibited the expression of IL-6, IL-1β, and BDNF.

While we showed the antinociceptive effect of CJE, (−)-epicatechin, and rutin against CCI-induced neuropathic pain and provided possible related mechanisms, this study has some limitations. For example, CJE and its active compounds were administered preoperatively in this study to prevent the development of pain hypersensitivity and inhibit microglia activation. The microglial inhibitor minocycline has been shown to prevent neuropathic pain, but did not reverse the developed pain sensitivity [73]. Therefore, further studies are needed to determine whether CJE and its active compounds could relieve established neuropathic pain and inhibit astrocyte activation, which is important in the late phase of neuropathic pain. Also, this report shows that, while CJE inhibited microglia in the spinal cord, the extent to which the neurons mediated its analgesic effect in the current model remains elusive.

## 5. Conclusions

In conclusion, our results demonstrate that CJE has antinociceptive effects on CCI-induced neuropathic pain by regulating maladaptive changes in the DRG and spinal cord. (−)-epicatechin and rutin are active compounds of CJE and attenuated pain sensitivity in CCI rats. CJE and its active compounds reduced MAPK activation, transcription factors, and nociceptive mediators in the DRG. Furthermore, microglial activation and neuroinflammation in the spinal cord are inhibited by CJE and its active compounds treatment. Thus, CJE, (−)-epicatechin, and rutin could be considered novel therapeutic candidates for peripheral neuropathic pain treatment.

## Figures and Tables

**Table 1:** Primer sequences for qRT-PCR.

Gene	Accession Number	Forward Primer	Reverse Primer
*Atf3*	NM_012912 XM_001068451	CGCCATCCAGAACAAGCA	TGAGCCCGGACGATACACG
*c-jun*	NM_021835	TGCAAAGATGGAAACGACCT	CCACTCTCGGACTGGAGGAA
*Cxcl14*	NM_001013137 XM_225162	GAGCACTGCCTGCATCCTAA	AAGGCTTTCGCACACAGCTA
*Tnf-α*	NM_012675	ACTGAACTTCGGGGTGATTG	GCTTGGTGGTTTGCTACGAC
*Aif1*	NM_017196 XM_346597	CAACAAGCACTTCCTCGATGATC	TGAAGGCCTCCAGTTTGGACT
*Il-6*	NM_012589	TCTCTCCGCAAGAGACTTCC	TCTTGGTCCTTAGCCACTCC
*Il-1β*	NM_031512	AAAGAAGAAGATGGAAAAGCGGTT	GGGAACTGTGCAGACTCAAACTC
*Bdnf*	NM_001270638	CTACGAGACCAAGTGCAATCC	AATCGCCAGCCAATTCTCTTT
*Gapdh*	NM_017008 XM_216453	GCACCGTCAAGGCTGAGAAC	TGGTGAAGACGCCAGTGGA

## Data Availability

Data is contained within the article.

## References

[1] Jensen T.S., Baron R., Haanpaa M., Kalso E., Loeser J.D., Rice A.S.C., Treede R.D. (2011). A new definition of neuropathic pain. Pain.

[2] Colloca L., Ludman T., Bouhassira D., Baron R., Dickenson A.H., Yarnitsky D., Freeman R., Truini A., Attal N., Finnerup N.B. (2017). Neuropathic pain. Nat. Rev. Dis. Primers.

[3] Van Hecke O., Austin S.K., Khan R.A., Smith B.H., Torrance N. (2014). Neuropathic pain in the general population: A systematic review of epidemiological studies. Pain.

[4] Gilron I., Baron R., Jensen T. (2015). Neuropathic pain: Principles of diagnosis and treatment. Mayo Clin. Proc..

[5] Schaefer C., Sadosky A., Mann R., Daniel S., Parsons B., Tuchman M., Anschel A., Stacey B.R., Nalamachu S., Nieshoff E. (2014). Pain severity and the economic burden of neuropathic pain in the United States: BEAT neuropathic pain observational study. Clin. Outcomes Res..

[6] Barohn R.J., Gajewski B., Pasnoor M., Brown A., Herbelin L.L., Kimminau K.S., Mudaranthakam D.P., Jawdat O., Dimachkie M.M., The Patient Assisted Intervention for Neuropathy: Comparison of Treatment in Real Life Situations (PAIN-CONTRoLS) Study Team (2021). Patient assisted intervention for neuropathy: Comparison of treatment in real life situations (PAIN-CONTRoLS): Bayesian adaptive comparative effectiveness randomized trial. JAMA Neurol..

[7] Krames E.S. (2014). The role of the dorsal root ganglion in the development of neuropathic pain. Pain Med..

[8] Lessans S., Lassiter C.B., Carozzi V., Heindel P., Semperboni S., Oggioni N., Chiorazzi A., Thompson C., Wagner M., Holden J. (2019). Global transcriptomic profile of dorsal root ganglion and physiological correlates of cisplatin-induced peripheral neuropathy. Nurs. Res..

[9] Martin S.L., Reid A.J., Verkhratsky A., Magnaghi V., Faroni A. (2019). Gene expression changes in dorsal root ganglia following peripheral nerve injury: Roles in inflammation, cell death and nociception. Neural Regen. Res..

[10] Chen G., Zhang Y.Q., Qadri Y.J., Serhan C.N., Ji R.R. (2018). Microglia in Pain: Detrimental and protective roles in pathogenesis and resolution of pain. Neuron.

[11] Ji R.R., Nackley A., Huh Y., Terrando N., Maixner W. (2018). Neuroinflammation and central sensitization in chronic and widespread pain. Anesthesiology.

[12] Tsuda M., Inoue K., Salter M.W. (2005). Neuropathic pain and spinal microglia: A big problem from molecules in “small” glia. Trends Neurosci..

[13] Jha M.K., Jeon S., Suk K. (2012). Glia as a link between neuroinflammation and neuropathic pain. Immune Netw..

[14] Tsuda M. (2016). Microglia in the spinal cord and neuropathic pain. J. Diabetes Investig..

[15] Hald A., Nedergaard S., Hansen R.R., Ding M., Heegaard A.M. (2009). Differential activation of spinal cord glial cells in murine models of neuropathic and cancer pain. Eur. J. Pain.

[16] Rojewska E., Korostynski M., Przewlocki R., Przewlocka B., Mika J. (2014). Expression profiling of genes modulated by minocycline in a rat model of neuropathic pain. Mol. Pain.

[17] Syngle A., Verma I., Krishan P., Garg N., Syngle V. (2014). Minocycline improves peripheral and autonomic neuropathy in type 2 diabetes: MIND study. Neurol. Sci..

[18] Bennett G.J., Xie Y.K. (1988). A peripheral mononeuropathy in rat that produces disorders of pain sensation like those seen in man. Pain.

[19] Dowdall T., Robinson I., Meert T.F. (2005). Comparison of five different rat models of peripheral nerve injury. Pharm. Biochem. Behav..

[20] Du H., Shi J., Wang M., An S., Guo X., Wang Z. (2018). Analyses of gene expression profiles in the rat dorsal horn of the spinal cord using RNA sequencing in chronic constriction injury rats. J. Neuroinflammation.

[21] Stephens K.E., Zhou W., Ji Z., Chen Z., He S., Ji H., Guan Y., Taverna S.D. (2019). Sex differences in gene regulation in the dorsal root ganglion after nerve injury. BMC Genom..

[22] Jeong C.-H., Kim J.H., Choi G.N., Kwak J.H., Kim D.-O., Heo H.J. (2010). Protective effects of extract with phenolics from camellia (*Camellia japonica*) leaf against oxidative stress-induced neurotoxicity. Food Sci. Biotechnol..

[23] Lee H.S., Choi J.H., Cui L., Li Y., Yang J.M., Yun J.J., Jung J.E., Choi W., Yoon K.C. (2017). Anti-Inflammatory and Antioxidative Effects of *Camellia japonica* on Human Corneal Epithelial Cells and Experimental Dry Eye: In Vivo and In Vitro Study. Investig. Ophthalmol. Vis. Sci..

[24] Yoon I.S., Park D.H., Kim J.E., Yoo J.C., Bae M.S., Oh D.S., Shim J.H., Choi C.Y., An K.W., Kim E.I. (2017). Identification of the biologically active constituents of *Camellia japonica* leaf and anti-hyperuricemic effect in vitro and in vivo. Int. J. Mol. Med..

[25] Majumder S., Ghosh A., Bhattacharya M. (2020). Natural anti-inflammatory terpenoids in *Camellia japonica* leaf and probable biosynthesis pathways of the metabolome. Bull. Natl. Res. Cent..

[26] Azuma C.M., Santos F.C.S.d., Lago J.H.G. (2011). Flavonoids and fatty acids of *Camellia japonica* leaves extract. Rev. Bras. Farmacogn..

[27] Van der Wal S., Cornelissen L., Behet M., Vaneker M., Steegers M., Vissers K. (2015). Behavior of neuropathic pain in mice following chronic constriction injury comparing silk and catgut ligatures. SpringerPlus.

[28] Chaplan S.R., Bach F.W., Pogrel J.W., Chung J.M., Yaksh T.L. (1994). Quantitative assessment of tactile allodynia in the rat paw. J. Neurosci. Methods.

[29] Cheng L., Duan B., Huang T., Zhang Y., Chen Y., Britz O., Garcia-Campmany L., Ren X., Vong L., Lowell B.B. (2017). Identification of spinal circuits involved in touch-evoked dynamic mechanical pain. Nat. Neurosci..

[30] Murthy S.E., Loud M.C., Daou I., Marshall K.L., Schwaller F., Kuhnemund J., Francisco A.G., Keenan W.T., Dubin A.E., Lewin G.R. (2018). The mechanosensitive ion channel Piezo2 mediates sensitivity to mechanical pain in mice. Sci. Transl. Med..

[31] Garrison S.R., Dietrich A., Stucky C.L. (2012). TRPC1 contributes to light-touch sensation and mechanical responses in low-threshold cutaneous sensory neurons. J. Neurophysiol..

[32] Boyd J.T., LoCoco P.M., Furr A.R., Bendele M.R., Tram M., Li Q., Chang F.-M., Colley M.E., Samenuk G.M., Arris D.A. (2021). Elevated dietary ω-6 polyunsaturated fatty acids induce reversible peripheral nerve dysfunction that exacerbates comorbid pain conditions. Nat. Metab..

[33] Zhao X., Tang Z., Zhang H., Atianjoh F.E., Zhao J.Y., Liang L., Wang W., Guan X., Kao S.C., Tiwari V. (2013). A long noncoding RNA contributes to neuropathic pain by silencing Kcna2 in primary afferent neurons. Nat. Neurosci..

[34] Attal N., Jazat F., Kayser V., Guilbaud G. (1990). Further evidence for ‘pain-related’ behaviours in a model of unilateral peripheral mononeuropathy. Pain.

[35] Miyakawa T., Terashima Y., Takebayashi T., Tanimoto K., Iwase T., Ogon I., Kobayashi T., Tohse N., Yamashita T. (2014). Transient receptor potential ankyrin 1 in spinal cord dorsal horn is involved in neuropathic pain in nerve root constriction rats. Mol. Pain.

[36] Park J., Kim Y.T. (2020). Erythronium japonicum alleviates inflammatory pain by inhibiting MAPK activation and by suppressing NF-kappaB activation via ERK/Nrf2/HO-1 signaling pathway. Antioxidants.

[37] Richner M., Jager S.B., Siupka P., Vaegter C.B. (2017). Hydraulic extrusion of the spinal cord and isolation of dorsal root ganglia in rodents. J. Vis. Exp..

[38] Ilari S., Giancotti L.A., Lauro F., Gliozzi M., Malafoglia V., Palma E., Tafani M., Russo M.A., Tomino C., Fini M. (2020). Natural antioxidant control of neuropathic pain-exploring the role of mitochondrial SIRT3 pathway. Antioxidants.

[39] Pietta P.G. (2000). Flavonoids as antioxidants. J. Nat. Prod..

[40] Jug U., Naumoska K., Vovk I. (2021). (−)-Epicatechin—An important contributor to the antioxidant activity of Japanese knotweed rhizome bark extract as determined by antioxidant activity-guided fractionation. Antioxidants.

[41] Magalingam K.B., Radhakrishnan A., Haleagrahara N. (2013). Rutin, a bioflavonoid antioxidant protects rat pheochromocytoma (PC-12) cells against 6-hydroxydopamine (6-OHDA)-induced neurotoxicity. Int. J. Mol. Med..

[42] Lopes Lda S., Marques R.B., Fernandes H.B., Pereira Sda S., Ayres M.C., Chaves M.H., Almeida F.R. (2012). Mechanisms of the antinociceptive action of (−) epicatechin obtained from the hydroalcoholic fraction of Combretum leprosum Mart & Eic in rodents. J. Biomed. Sci..

[43] Quinonez-Bastidas G.N., Pineda-Farias J.B., Flores-Murrieta F.J., Rodriguez-Silverio J., Reyes-Garcia J.G., Godinez-Chaparro B., Granados-Soto V., Rocha-Gonzalez H.I. (2018). Antinociceptive effect of (−)-epicatechin in inflammatory and neuropathic pain in rats. Behav. Pharmacol..

[44] Quinonez-Bastidas G.N., Cervantes-Duran C., Rocha-Gonzalez H.I., Murbartian J., Granados-Soto V. (2013). Analysis of the mechanisms underlying the antinociceptive effect of epicatechin in diabetic rats. Life Sci..

[45] Al-Enazi M.M. (2013). Ameliorative potential of rutin on streptozotocin-induced neuropathic pain in rat. Afr. J. Pharm. Pharmacol..

[46] Azevedo M.I., Pereira A.F., Nogueira R.B., Rolim F.E., Brito G.A., Wong D.V.T., Lima-Júnior R.C., de Albuquerque Ribeiro R., Vale M.L. (2013). The antioxidant effects of the flavonoids rutin and quercetin inhibit oxaliplatin-induced chronic painful peripheral neuropathy. Mol. Pain.

[47] Lee G.H., Kim S.S. (2016). Therapeutic strategies for neuropathic pain: Potential application of pharmacosynthetics and optogenetics. Mediat. Inflamm..

[48] Zhang W., Liu H.T. (2002). MAPK signal pathways in the regulation of cell proliferation in mammalian cells. Cell Res..

[49] Qu Y.J., Jia L., Zhang X., Wei H., Yue S.W. (2016). MAPK pathways are involved in neuropathic pain in rats with chronic compression of the dorsal root ganglion. Evid.-Based Complement. Altern. Med..

[50] Chen Q., Kong L., Xu Z., Cao N., Tang X., Gao R., Zhang J., Deng S., Tan C., Zhang M. (2021). The Role of TMEM16A/ERK/NK-1 signaling in dorsal root ganglia neurons in the development of neuropathic pain induced by Spared Nerve Injury (SNI). Mol. Neurobiol..

[51] Dai W.L., Yan B., Bao Y.N., Fan J.F., Liu J.H. (2020). Suppression of peripheral NGF attenuates neuropathic pain induced by chronic constriction injury through the TAK1-MAPK/NF-kappaB signaling pathways. Cell Commun. Signal..

[52] Pokhilko A., Nash A., Cader M.Z. (2020). Common transcriptional signatures of neuropathic pain. Pain.

[53] Sisignano M., Baron R., Scholich K., Geisslinger G. (2014). Mechanism-based treatment for chemotherapy-induced peripheral neuropathic pain. Nat. Rev. Neurol..

[54] Rivat C., Sar C., Mechaly I., Leyris J.P., Diouloufet L., Sonrier C., Philipson Y., Lucas O., Mallie S., Jouvenel A. (2018). Inhibition of neuronal FLT3 receptor tyrosine kinase alleviates peripheral neuropathic pain in mice. Nat. Commun..

[55] Kan H.W., Chang C.H., Chang Y.S., Ko Y.T., Hsieh Y.L. (2021). Genetic loss-of-function of activating transcription factor 3 but not C-type lectin member 5A prevents diabetic peripheral neuropathy. Lab. Investig..

[56] Son S.J., Lee K.M., Jeon S.M., Park E.S., Park K.M., Cho H.J. (2007). Activation of transcription factor c-jun in dorsal root ganglia induces VIP and NPY upregulation and contributes to the pathogenesis of neuropathic pain. Exp. Neurol..

[57] Wlaschin J.J., Gluski J.M., Nguyen E., Silberberg H., Thompson J.H., Chesler A.T., Le Pichon C.E. (2018). Dual leucine zipper kinase is required for mechanical allodynia and microgliosis after nerve injury. Elife.

[58] Ellis A., Bennett D.L. (2013). Neuroinflammation and the generation of neuropathic pain. Br. J. Anaesth..

[59] Sacerdote P., Franchi S., Trovato A.E., Valsecchi A.E., Panerai A.E., Colleoni M. (2008). Transient early expression of TNF-alpha in sciatic nerve and dorsal root ganglia in a mouse model of painful peripheral neuropathy. Neurosci. Lett..

[60] Ogawa N., Kawai H., Terashima T., Kojima H., Oka K., Chan L., Maegawa H. (2014). Gene therapy for neuropathic pain by silencing of TNF-alpha expression with lentiviral vectors targeting the dorsal root ganglion in mice. PLoS ONE.

[61] Andrade P., Hoogland G., Del Rosario J.S., Steinbusch H.W., Visser-Vandewalle V., Daemen M.A. (2014). Tumor necrosis factor-alpha inhibitors alleviation of experimentally induced neuropathic pain is associated with modulation of TNF receptor expression. J. Neurosci. Res..

[62] Strong J.A., Xie W., Coyle D.E., Zhang J.M. (2012). Microarray analysis of rat sensory ganglia after local inflammation implicates novel cytokines in pain. PLoS ONE.

[63] Poisbeau P., Aouad M., Gazzo G., Lacaud A., Kemmel V., Landel V., Lelievre V., Feron F. (2019). Cholecalciferol (Vitamin D3) reduces rat neuropathic pain by modulating opioid signaling. Mol. Neurobiol..

[64] Kawasaki Y., Xu Z.Z., Wang X., Park J.Y., Zhuang Z.Y., Tan P.H., Gao Y.J., Roy K., Corfas G., Lo E.H. (2008). Distinct roles of matrix metalloproteases in the early- and late-phase development of neuropathic pain. Nat. Med..

[65] Illias A.M., Gist A.C., Zhang H., Kosturakis A.K., Dougherty P.M. (2018). Chemokine CCL2 and its receptor CCR2 in the dorsal root ganglion contribute to oxaliplatin-induced mechanical hypersensitivity. Pain.

[66] Wen J., Jones M., Tanaka M., Selvaraj P., Symes A.J., Cox B., Zhang Y. (2018). WWL70 protects against chronic constriction injury-induced neuropathic pain in mice by cannabinoid receptor-independent mechanisms. J. Neuroinflammation.

[67] Yu X., Liu H., Hamel K.A., Morvan M.G., Yu S., Leff J., Guan Z., Braz J.M., Basbaum A.I. (2020). Dorsal root ganglion macrophages contribute to both the initiation and persistence of neuropathic pain. Nat. Commun..

[68] Zhao L., Song C., Huang Y., Lei W., Sun J. (2020). MMP-9 regulates CX3CL1/CX3CR1 in the early phase of neuropathic pain in chronic sciatic nerve constriction injury (CCI) rats. Ann. Palliat. Med..

[69] Amin B., Noorani R., Marjan Razavi B., Hosseinzadeh H. (2018). The effect of ethanolic extract of lippia citriodora on rats with chronic constriction injury of neuropathic pain. Cell J..

[70] Yao Y., Tan Y.H., Light A.R., Mao J., Yu A.C., Fu K.Y. (2016). Alendronate attenuates spinal microglial activation and neuropathic pain. J. Pain.

[71] Reeve A.J., Patel S., Fox A., Walker K., Urban L. (2000). Intrathecally administered endotoxin or cytokines produce allodynia, hyperalgesia and changes in spinal cord neuronal responses to nociceptive stimuli in the rat. Eur. J. Pain.

[72] Wolf G., Gabay E., Tal M., Yirmiya R., Shavit Y. (2006). Genetic impairment of interleukin-1 signaling attenuates neuropathic pain, autotomy, and spontaneous ectopic neuronal activity, following nerve injury in mice. Pain.

[73] Shibata K., Sugawara T., Fujishita K., Shinozaki Y., Matsukawa T., Suzuki T., Koizumi S. (2011). The astrocyte-targeted therapy by Bushi for the neuropathic pain in mice. PLoS ONE.

